# Lipids in kidney diseases: from systemic imbalance to intrarenal alterations of cellular lipid metabolism in rare and common kidney diseases

**DOI:** 10.1007/s00109-026-02654-0

**Published:** 2026-02-20

**Authors:** Carola Garavaglia, Alice Ossoli, Monica Gomaraschi

**Affiliations:** Centro E. Grossi Paoletti, Dipartimento Di Scienze Farmacologiche E Biomolecolari, Università Degli Studi Di Milano, Via Balzaretti 9, 20133 Milan, Italy

**Keywords:** Lipid metabolism, Lipoproteins, Chronic kidney disease, Diabetes, Inborn errors of metabolism, Glomerulosclerosis, Interstitial fibrosis

## Abstract

Chronic kidney disease (CKD) is expected to be the fifth cause of global mortality by 2040. Modifiable and non-modifiable risk factors for CKD range from metabolic conditions, such as diabetes or obesity, to genetic defects. Regardless of the triggering factor, irreversible injury and loss of kidney cells result in the decline of kidney function. Renal damage often occurs through common signaling pathways promoting inflammation, oxidative stress, organelle dysfunction, complement activation, apoptosis, and fibrogenesis. Lipotoxicity is emerging as a key player in CKD of both metabolic and non-metabolic origin. Here, we provide an overview of the mechanisms beyond renal lipid accumulation in CKD, ranging from systemic imbalance of lipid/lipoprotein metabolism to alterations of cell lipid uptake, synthesis, and disposal. Moreover, the impact of lipid accumulation in glomerular and tubular cells is addressed. Several lipid species were shown to accumulate in renal cells, from cholesterol and fatty acids to sphingolipids; they can affect cell viability and function not only as structural components of biological membranes or energetic substrates but also as bioactive molecules able to modulate multiple signaling pathways. The possible role of established and novel approaches to correct systemic or local lipid imbalance in the management of CKD is discussed.

## Introduction

The kidney plays a central role in maintaining the body’s homeostasis, and while its functions are primarily associated with filtration, excretion, and regulation of electrolyte balance, recent advances in metabolic research have highlighted the kidney’s critical involvement in lipid metabolism. Lipid homeostasis in the kidney is essential for the organ’s structural integrity, function, and adaptation to changing metabolic conditions [1]. Lipids, including fatty acids (FAs), cholesterol, phospholipids (PL), and many other minor species, serve as essential bioactive molecules involved not only in energy storage and biological membrane structure, but also in intracellular and intercellular signaling by regulating vital processes such as inflammation, oxidative stress, cell proliferation, or apoptosis. Importantly, the kidney also plays a pivotal role in lipid clearance, maintaining systemic lipid balance and preventing the accumulation of potentially harmful lipid species in the bloodstream. The kidney can use both exogenous and endogenous sources of lipids, thus allowing it to maintain energy balance even in periods of nutritional fluctuation, fasting, or metabolic stress [2, 3]. Several studies performed in vitro and in animal models have underscored the importance of lipid dysregulation in the pathogenesis of various kidney diseases, showing a link between kidney dysfunction and renal lipid accumulation in many metabolic diseases, as well as in other forms of chronic kidney disease, acute renal injury, and aging [4]. Interestingly, renal lipid accumulation has been demonstrated even in conditions that are not associated with systemic dyslipidemias, thus suggesting that intrarenal alterations of cellular lipid metabolism can independently occur during kidney disease development and progression [5].

This review aims to provide an overview of the molecular pathways governing lipid synthesis, uptake, storage, and disposal in the kidneys, their alterations in metabolic and non-metabolic pathological conditions, and the underlying mechanisms of damage.

## Lipids in the kidney: a role in energy production and beyond

**Fig. 1:**
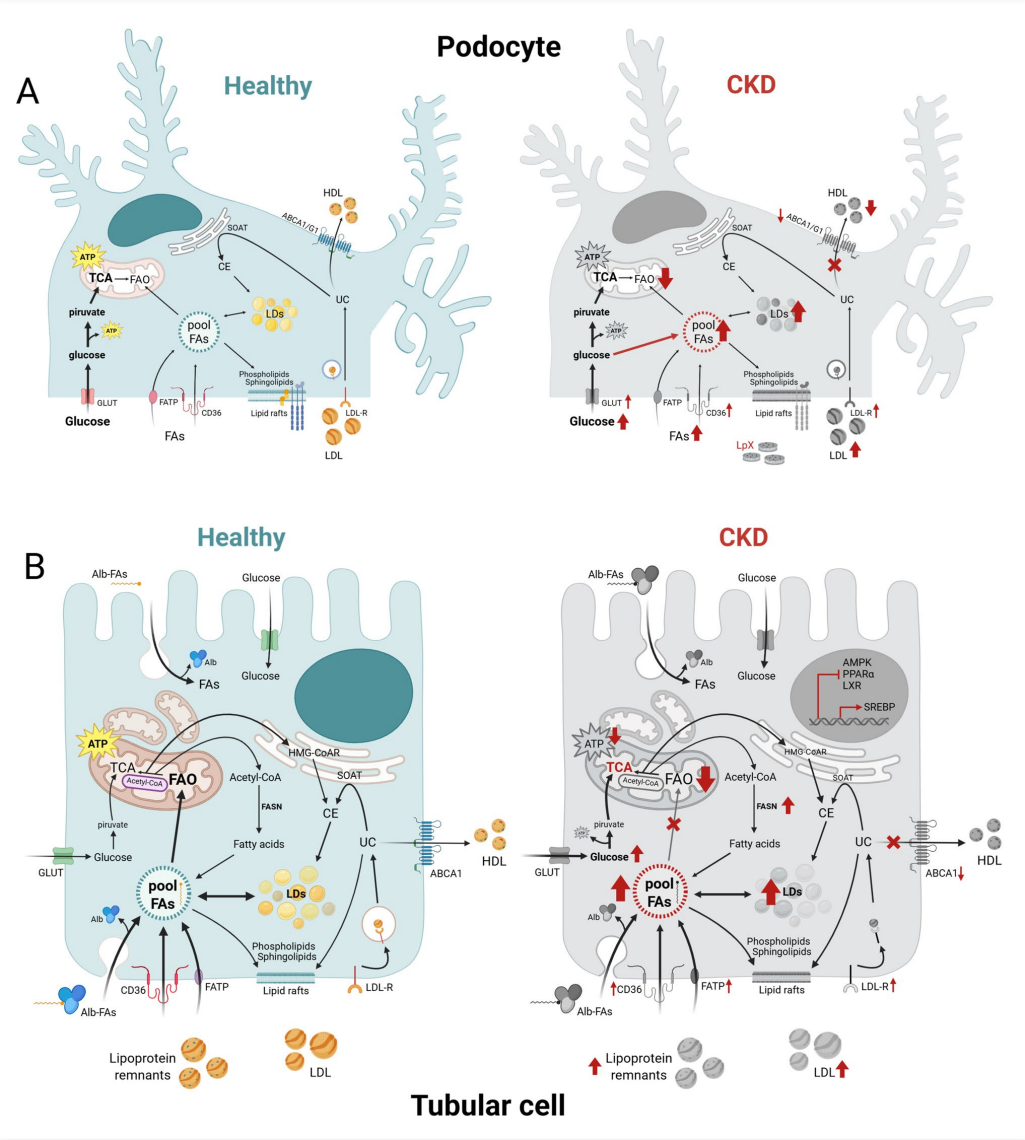
Energetic metabolism of renal cells and its pathological alterations. Schematic representation of energetic metabolism in podocytes (**A**) and tubular epithelial cells (TECs) (**B**). Healthy cells are depicted in light blue, while pathological changes occurring during CKD are depicted in gray. Red arrows indicate upregulated or downregulated processes. Healthy podocytes mainly rely on glycolysis for their energetic demand, while TECs use mitochondrial fatty acid oxidation (FAO) for ATP production. In both cells, lipids can be taken up by receptor-mediated endocytosis of circulating lipoproteins or locally synthesized. Lipid disposal occurs through transporter-mediated efflux to extracellular acceptors, as HDL. In CKD, lipids accumulate into cytosolic lipid droplets (LD) and in organelles as the mitochondria, where they cause defective FAO. This accumulation is driven by an increased uptake of circulating lipoproteins and free FA coupled with an impairment of lipid efflux. When cellular glucose is elevated, acetyl-CoA is used for FA synthesis as well. Please refer to the abbreviation list and the main text for further details. Created with BioRender

The kidneys receive 25% of the cardiac output to maintain fluid homeostasis by filtering waste from the blood, reabsorbing essential nutrients, regulating electrolyte balance, controlling blood pH and pressure, and producing hormones, thanks to their highly specialized structure and energy-demanding processes. Indeed, while blood filtration is a passive process driven by glomerular hydrostatic pressure, tubular reabsorption and secretion rely on active transport, which is energetically costly. Consequently, the different kidney compartments have distinct energetic requirements and substrate preferences for ATP production [6] (Fig. 1). Glomeruli are composed of podocytes, mesangial cells, and endothelial cells that mainly rely on glycolysis for their limited energetic demand, with mitochondrial FA oxidation (FAO) as an alternative energy source during metabolic stress, such as when glucose availability is low. On the contrary, tubular epithelial cells (TECs), especially those located in the proximal tubule (PTECs), are more energy-demanding due to the high density of active transport systems, such as the Na^+^/K^+^ ATPase. Consequently, they are enriched in mitochondria and predominantly use FAO for ATP production. Indeed, although PTECs reabsorb and transport hundreds of grams of glucose each day and are also capable of gluconeogenesis during starvation or metabolic stress, they do not typically rely on glucose as their main energy source, being FAO more efficient in terms of ATP molecules produced per carbon unit. However, when mitochondrial respiration is inhibited, PTECs can shift to glycolysis to meet their metabolic demand [6, 7].

### Fatty acids

FAs play a key role in renal physiology not only as substrates for FAO, but also as structural and signaling molecules. As building blocks for PL, FAs are the main components of all biological membranes, whose fluidity also depends on the balance between saturated and unsaturated FAs in the PL bilayer. In addition, FAs and their metabolites can activate various nuclear receptors and transcription factors, thus modulating the expression of genes involved not only in lipid and glucose metabolism, but also in inflammation and kidney function [8, 9]. As depicted in Fig. 1, renal cells can take up FAs from the circulation through receptor-mediated endocytosis of lipoproteins followed by the hydrolysis of PL, triglycerides (TG), and cholesteryl esters (CE) in the lysosomes. Indeed, both tubular and glomerular cells express scavenger receptors, such as CD36, and members of the low-density lipoprotein receptor family, such as the LDL-receptor (LDL-R) and LDL-related protein receptors (LRPs) [10]. Albumin-bound FAs in the circulation or in the glomerular filtrate can enter renal cells by LRP2/megalin-mediated endocytosis and lysosomal hydrolysis as well. In addition, fatty acid transport proteins (FATP) and fatty acid-binding proteins (FABP) are expressed especially on PTECs and mediate the uptake of free FAs (FFAs) [11]. FAs can also be synthesized locally from glucose by de novo *lipogenesis* (DNL) [4]. FAO is tightly regulated by a complex network of interrelated enzymes and transcription factors like AMP-activated protein kinase (AMPK), sterol regulatory element-binding proteins (SREBPs), carbohydrate-response element-binding protein (ChREBP), peroxisome proliferator–activated receptor alpha (PPARα), liver X receptors (LXRs), farnesoid X receptors (FXRs), and estrogen-related receptor alpha (ESRRα) [9, 12, 13]. Acetyl-CoA exceeding cell energetic demand or mitochondrial oxidative capacity can be moved back to the cytosol and used for DNL of FAs or cholesterol. Due to their direct cytotoxicity, when FFAs are not promptly used, they are converted into TG and stored in cytoplasmic lipid droplets (LD). These LDs can undergo hydrolysis by neutral lipases in the cytosol or acid lipases in the lysosomes after the activation of autophagy to provide FFAs when needed. Autophagic flux is physiologically elevated in PTECs, but also in podocytes, which are terminally differentiated epithelial cells: the degradation of cellular components and an effective recycling of the slit diaphragm proteins are needed to maintain the structural and functional integrity of podocytes [4, 14].

### Cholesterol

Cholesterol is another key component of biological membranes. It contributes not only to membrane fluidity but also to cell signaling, especially within lipid rafts which represent a platform for the efficient activation of several transmembrane receptors [13]. In podocytes, the assembly of the slit diaphragm that governs glomerular filtration occurs within the lipid rafts [15]. In addition, cholesterol can be converted into other biologically active molecules as steroid hormones and oxysterols. Mineralocorticoids regulate salt and water balance, while glucocorticoids modulate inflammation and stress responses [16, 17]. Oxysterols can activate nuclear receptors as the LXRs and regulate inflammation, apoptosis, and cell survival [18]. As for FAs, cholesterol can be locally synthesized or taken up from circulation (Fig. 1). Cholesterol synthesis through the mevalonate pathway is limited in the kidney and largely regulated by SREBPs, whose activation is repressed by PPARs, LXRs, and FXRs. Receptor-mediated endocytosis of lipoproteins occurs as described above and cholesterol generated in the lysosomes by lysosomal acid lipase is transported by Niemann–Pick C1 and C2 proteins (NPC1 and NPC2) into the cytosol and to other cellular compartments as the endoplasmic reticulum (ER), membranes, and mitochondria [19]. Kidney cells can dispose of excess cholesterol by efflux through the ATP-binding cassette (ABC) transporters A1 and G1; these transporters are induced by LXRs and mediate cholesterol and PL efflux toward extracellular acceptors as high-density lipoproteins (HDL) [20]. In addition, cholesterol can be esterified by sterol O-acyltransferase (SOAT) and the generated CE can be stored in LDs as well [21].

### Other lipids

Among other minor lipid species, sphingolipids are of particular interest. As for cholesterol, their content is high within lipid rafts, but since many of them are biologically very active, their intracellular concentration must be finely tuned, especially in the glomeruli. This is also true for ceramides, which are generated during sphingolipid catabolism in the lysosomes, as from sphingomyelin (SM) via acid sphingomyelinase (ASM) [15].

Physiologically, renal lipid content is relatively low, but dysregulation of both systemic and intracellular lipid homeostasis can lead to lipid accumulation in renal glomeruli and tubules [13], which can directly or indirectly contribute to the pathogenesis and progression of kidney disease [22]. Lipid accumulation can be the consequence of increased uptake or synthesis, or decreased disposal capacity, including catabolism and efflux (Fig. 1). As a protective mechanism, excessive lipids are stored in LDs in order to prevent their accumulation in various organelles such as the ER, lysosomes, and mitochondria, where they can trigger organelle dysfunction [4].

## Chronic kidney disease

Chronic kidney disease (CKD) is the tenth leading cause of global mortality in 2019, and it is expected to be the fifth cause by 2040 with a global prevalence in 2022 estimated at ~ 850 million people [23]. Risk factors for CKD vary with age and world region and are classified as modifiable and non-modifiable. Modifiable risk factors include the presence of active kidney disease, described as recurrent episodes of acute kidney injury, overweight and obesity, diabetes mellitus, arterial hypertension, exposure to intrinsic/extrinsic nephrotoxins or environmental stress factors, and pregnancy. Among non-modifiable risk factors, beyond sex and age, glomerulonephritis, polycystic kidney disease, congenital anomalies of renal development, and the negative effects of variants in the *APOL1*, *PKD1*, or *PKD2* (autosomal dominant polycystic kidney disease) and *COL4A3*, *COL4A4*, or *COL4A5* (Alport syndrome) genes were described [24].

Regardless of the triggering factor, the irreversible injury and loss of kidney cells and nephrons result in the decline of kidney function. The workload of the remaining cells increases, but when further compensatory adaptation exceeds nephron capacity, the disease evolves toward CKD [24]. CKD is characterized by structural abnormalities, such as glomerulosclerosis and/or interstitial fibrosis, and by functional alterations, such as reduced excretory capacity, impaired glomerular barrier function, and blood electrolyte disturbances. Fibrosis is the final pathological process of CKD and is defined as excessive deposition of extracellular matrix, which disrupts and replaces the functional parenchyma, leading to organ failure. Kidney fibrosis is identified by tubular atrophy, interstitial chronic inflammation and fibrogenesis, glomerulosclerosis, and vascular rarefaction [25].

CKD classification and staging take into account the cause of kidney disease and the severity of kidney dysfunction, using glomerular filtration rate (GFR) as an indicator of the excretory capacity of the kidneys, and the extent of albuminuria as a marker of glomerular barrier function. CKD severely affects human physiology and, in particular, the cardiovascular system. Indeed, CKD patients display an elevated incidence of major adverse cardiac events with the highest risk for patients undergoing dialysis [26]. Blood volume expansion and hypertension are frequently the first signs of CKD. They are the consequence of reduced sodium excretion and increased sympathetic nervous system and renin–angiotensin–aldosterone axis (RAAS) activation to maintain a normal GFR when kidney function is reduced [27]. A concomitant reduction of potassium excretion may lead to hyperkaliemia, a risk factor for arrhythmia-related sudden cardiac death [28]. Moreover, when GFR decreases below 40–50 ml/min/1.73 m^2^, metabolic acidosis arises and contributes to bone demineralization, loss of muscle mass, and CKD progression [29]. CKD is also associated with secondary immunodeficiency due to hyperuricemia, anemia due to impaired erythropoietin secretion, cognitive dysfunction, and loss of endocrine homeostasis [30, 31].

## Lipid accumulation due to systemic imbalance of lipid metabolism

**Fig. 2:**
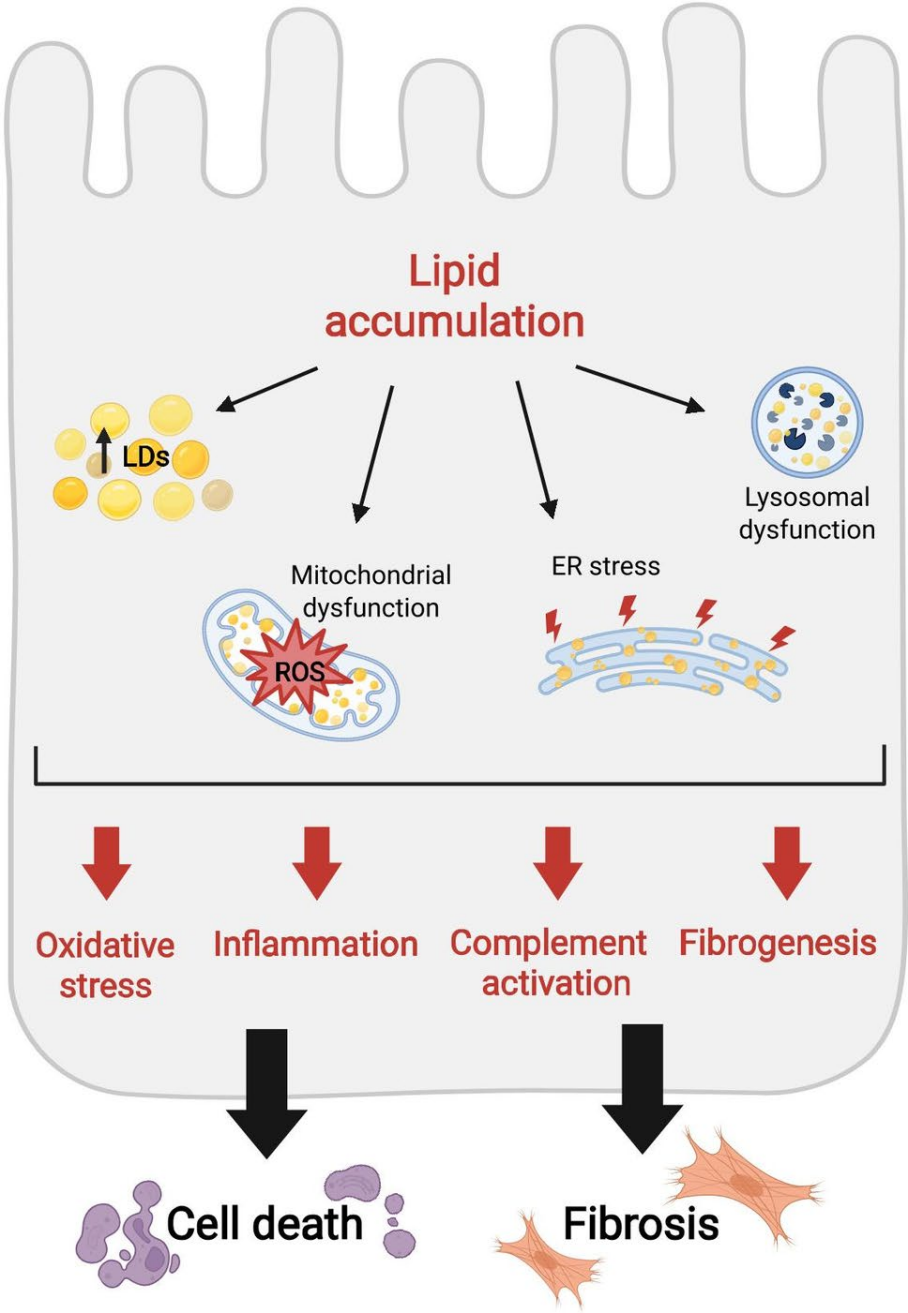
Mechanisms of renal lipotoxicity. Excessive lipids accumulate not only into cytosolic lipid droplets (LD) but also in the endoplasmic reticulum (ER), the lysosomes, and mitochondria. The consequent organelle dysfunction could result in increased oxidative stress and in the activation of inflammation, the complement cascade, and of pro-fibrogenic pathways, thus leading to renal cell death and fibrosis. Please refer to the abbreviation list and the main text for further details. Created with BioRender

Lipid deposition in glomeruli and tubules has been observed in genetic and non-genetic forms of kidney disease [32]. It could be due to a systemic imbalance of lipid metabolism and/or to altered intracellular lipid homeostasis in the kidney compartment (Fig. 1). In both cases, the resulting lipotoxic cascade can contribute to kidney disease development and progression, although the nature of this link is still not completely understood (Fig. 2).

### Diabetic dyslipidemia

Among non-genetic forms of CKD, DKD is the one for which lipotoxicity is a well-recognized driver of injury. However, its relevance for human pathology is still underestimated since renal biopsies are not routinely performed in diabetic patients and samples are not processed for lipids [33]. Diabetic patients usually display a peculiar lipid profile, called diabetic dyslipidemia, which is characterized by fasting and postprandial hypertriglyceridemia, reduced plasma levels of HDL-cholesterol, while LDL-cholesterol levels are often normal but with a prevalence of small dense particles [34]. Hypertriglyceridemia is due to an increased secretion of TG-containing lipoproteins by both enterocytes and hepatocytes coupled with their decreased catabolism. These lipoproteins can be taken up by renal cells as described above. Moreover, insulin resistance promotes sustained lipolysis in the adipocytes which causes increased circulating levels of FFAs [35]. This systemic abundance of FFAs can cause their accumulation in the kidney, with a cytotoxicity that is proportional to acyl chain length and carbon bond saturation [36, 37]. Consistently, plasma levels of saturated FFAs as palmitate and stearate are increased in diabetic patients and correlate with CKD stage [38, 39]. Saturated FFAs, especially palmitate, are not readily incorporated into TG and LDs, but shunted to the generation of ceramide in the ER and of reactive oxygen species (ROS). Altogether, these events can trigger apoptosis of renal cells [40] (Fig. 2).

### Genetic dyslipidemias

Lipid accumulation in the kidneys can also be driven by mutations in genes coding for key regulators of systemic lipid metabolism, such as LCAT and apolipoprotein E (apoE). LCAT is the enzyme responsible for cholesterol esterification in plasma. Mutations abolishing LCAT activity severely affect lipid and lipoprotein profiles: circulating lipoproteins are enriched in unesterified cholesterol, the levels of HDL are reduced with a predominance of native discoidal particles, and an abnormal lipoprotein, called lipoprotein X (LpX, Fig. 1), is often detected in the plasma of affected patients [41]. LpX is a multilamellar vesicle made of PLs and unesterified cholesterol and devoid of hydrophobic core lipids. The first cause of morbidity and mortality in patients with a complete lack of LCAT activity is renal disease [42]. Proteinuria can appear as early as in the second decade of life, usually progressing to CKD by the fourth decade of life [43]. However, the development and progression of kidney disease in LCAT-deficient patients are unpredictable and variable, also within the same family, suggesting a role for genetics and environment in kidney decline [44]. Kidney biopsies clearly show the hallmarks of glomerulosclerosis with histopathologic changes such as mesangial expansion, irregular thickening of the glomerular capillary walls, and increased mesangial cellularity [45]. Unesterified cholesterol and PLs accumulate in the glomeruli, inducing vacuolization of the basement membrane and conferring a “foamy” appearance [45]. Other peculiar alterations include the modification of podocyte structure with fused endothelial processes, the presence of IgM deposits, and C3 in the capillary loops, the mesangium, and arteriolar walls [46, 47]. Although the pathogenesis of renal disease in LCAT deficiency has not been fully clarified yet, the direct role of LpX has been demonstrated in mice. After intravenous administration of synthetic LpX, LCAT knockout mice developed proteinuria and renal histological hallmarks [48]. In humans, LpX has been detected in the capillary loops of the glomerulus, where it induces vascular and endothelial damage [47]. In vitro, abnormal lipoproteins isolated from the plasma of LCAT-deficient patients, in particular LpX and HDL, were shown to trigger oxidative stress in renal cells, culminating in podocyte and tubular cell apoptosis. These effects are likely due to the enrichment in unesterified cholesterol and ceramides, especially in the HDL fraction [49].

Another rare condition in which kidney disease is associated with lipid accumulation and the presence of LpX is Alagille syndrome (ALGS). ALGS is an autosomal dominant disease characterized by abnormal development of intrahepatic bile ducts, heart, arteries, and kidneys due to disrupted Notch signaling [50]. Renal involvement has been reported in 40–70% of Alagille cases, primarily as developmental defects. However, renal histopathology described in ALGS confirms the presence of mesangiolipidosis in some cases, with lipid deposit predominately in the mesangium but also in the basement membrane, and the appearance of FSGS [51, 52]. Proteinuria is likely attributable to significant lipid deposition in the glomerular basement membrane, which may contribute to podocyte injury and, over time, the development of FSGS [52]. ALGS patients often display a dyslipidemia characterized by the appearance of LpX in the circulation, which may contribute to lipid deposition in kidneys [53].

Mutations in the gene coding for apoE have also been associated with renal disease. ApoE is one of the key protein components of several circulating lipoproteins [54]. The *APOE* gene is highly polymorphic, and three major isoforms (E2, E3, and E4) have been described. Homozygosity for apoE2 causes familial type III hyperlipidemia, which is characterized by the accumulation of chylomicron remnants and VLDL remnants in the circulation; consequently, affected patients display similarly elevated plasma total cholesterol and TG levels (usually above 300 mg/dL) [55]. Rarer mutations leading to apoE deficiency and severe type III hyperlipidemia have also been described. Glomerulopathy has been reported in apoE2 homozygotes: circulating lipoproteins are taken up by mesangial cells and by macrophages infiltrating the mesangium, contributing to the development of glomerulosclerosis [56]. Interestingly, other mutations in the *APOE* gene can cause another type of renal disease, named lipoprotein glomerulopathy (LPG), even in the absence of a clear dyslipidemic phenotype [57]. These apoE mutants have low affinity for LDL-R/LRPs and defective folding, leading to protein instability and propensity to aggregation [57, 58]. Indeed, human LPG is characterized by the presence of dilated glomerular capillaries occluded by clots of lamellar lipoproteins containing mutated apoE, resulting in ischemic glomerular damage and proteinuria gradually progressing to ESRD [59]. Intraluminal lipoprotein precipitation in the glomeruli is likely due to their defective receptor-mediated uptake by renal cells and by infiltrating monocytes/macrophages, coupled with apoE mutant instability in the renal microenvironment and with the tortuous structure of glomerular capillaries [55, 58, 60].

## Lipid accumulation due to defective renal cell lipid homeostasis

Alterations of cellular lipid metabolism in both glomerular and tubular cells leading to the accumulation of various lipid species have been described in several conditions. As stated above, in patients with diabetes, but also with obesity or metabolic syndrome, renal lipid accumulation is driven by systemic dyslipidemia; however, it is also supported by specific changes in the expression or activity of key players in intracellular lipid metabolism in PTECs and podocytes (Fig. 1).

### Changes in lipid uptake, synthesis, and disposal

In renal biopsies of patients with DKD, a general overactivation of lipid uptake and synthesis coupled with a reduction of lipid efflux has been described [13, 61] (Fig. 1). The increased expression of receptors such as LDL-R and CD36, and of FATP2 allows the uptake of the elevated circulating levels of TG-rich lipoproteins and free or albumin-bound FAs in diabetic patients [11, 62]. In addition, since the cellular content of glucose is elevated, DNL is increased as well. In parallel, cholesterol and PL efflux are impaired due to decreased expression of ABC transporters [63]. Interestingly, similar alterations were also observed in non-metabolic forms of CKD, as in the renal cortex of mice with Alport Syndrome [64].

These changes have been ascribed to the reduced activation of AMPK, PPARα and LXRs, and increased activity of SREPBs, as shown in animal models of CKD [13, 63, 65, 66] (Fig. 1). Regarding SREPBs, insulin could directly increase their expression, while ER stress induced by hyperglycemia could favor their activation [67]. Since FFAs and UC are cytotoxic, they are quickly converted into TG and CE and stored in cytosolic LDs. Thus, the presence of LDs in renal cells is per se an indication of an altered metabolic homeostasis. Moreover, LDs are very dynamic and can establish contact sites with other organelles such as the ER, lysosomes, mitochondria and peroxisomes [68]. Through these membrane-membrane connections lipids can be transferred from LDs to the organelles, especially when storage capacity of LDs is overcome. Lipid accumulation triggers ER stress, mitochondrial and lysosomal dysfunction [69] (Fig. 2). Dysfunctional mitochondria have defective FAO, increased ROS production and trigger apoptosis. Besides evidence in animal models, this metabolic reprogramming of mitochondria driven by AMPK and PPARα inhibition has been confirmed in the renal cortex of patients with autosomal dominant polycystic kidney disease due to mutations in the *PKD1* gene [70, 71]. Lysosomes, in which cholesterol accumulates also due to NPC1 and NPC2 downregulation, show impaired acidification and conversion into multilamellar bodies; dysfunctional lysosomes blunt the autophagic flux, which is key for cell viability of both PTECs and podocytes [72]. The final outcome is cytoskeleton derangement, foot process effacement and cell death in podocytes, and induction of fibrosis in the tubules [65, 73]. Since the lysosome is responsible for the catabolism of sphingolipids, lysosomal dysfunction results in the accumulation of other lipid species, like SM and ceramides [74]. Altogether, lipids can induce inflammasome activation by acting as damage-associated molecular patterns (DAMPs) [75].

### Changes in lysosomal catabolism of lipids

Accumulation of lipid species, such as cholesterol, sphingolipids, and glycolipids, in renal cells has been described and associated with the development of kidney disease in various forms of lysosomal storage disorders (LSD). In particular, renal involvement is common in Fabry disease, and renal failure is one of the major causes of death in affected patients [76]. Fabry disease is an X-linked LSD due to defective lysosomal α-galactosidase A, the enzyme responsible for the hydrolysis of galactose from sphingolipids; specifically, it results in the progressive deposition of glycosphingolipids with terminal α-galactosyl moieties, especially globotriaosylceramide and its metabolite globotriaosylsphingosine, in the lysosomes. This accumulation also occurs in the kidney: all renal cells are affected, but podocytes seem to be particularly sensitive. In human podocytes, the accumulation of glycosphingolipids triggers inflammation and oxidative stress, impairs autophagic flux, and induces cell apoptosis, leading to the development of FSGS, interstitial fibrosis, and tubular atrophy [77–79]. Microscopically, lysosomes are converted into lamellar bodies, which are called zebra bodies, enriched in PL and sphingolipids. In affected patients, enzyme replacement therapy is effective in clearing lipid deposits, but it affects renal disease progression only when organ involvement is not advanced [80]. Fewer evidence is available for other lipid-related LSD as Niemann–Pick disease, Gaucher disease, Farber disease and mucolipidosis. Niemann–Pick Disease type A or B is due to defective lysosomal ASM, the enzyme responsible for the catabolism of SM into ceramide and phosphorylcholine and coded by the sphingomyelin phosphodiesterase 1 gene (*SMPD1*). Type A is a fatal disorder of infancy leading to death by 3 years of age, while type B has a wide phenotypic expression with little or no neurologic involvement and survives into adulthood. Other than SM, cholesterol and lyso-SM accumulate in the lysosomal compartment as well. Most patients have an atherogenic lipid profile with low levels of HDL-C, elevated total and LDL-C, and TGs. Cases of sphingolipid accumulation in patients’ kidneys are rare but confirmed in the *SMPD1* KO mice [81, 82]. No available data regarding the impact of such changes on renal function and related mechanisms are available to date. Niemann–Pick type C is mainly due to mutation in the *NPC1* gene and is a fatal autosomal recessive neurovisceral disease; renal involvement in humans is still unclear. Dysfunctional NPC1 does not allow the egress of unesterified cholesterol and SM from the lysosomes. Recently, pathological changes in kidney morphology were shown in a mouse model of the disease; in particular, lack of NPC1 induced significant vacuolization, with abnormal activation of the Wnt signaling pathway leading to apoptosis and fibrosis in tubular epithelial cells [83]. Gaucher disease, the most common LSD, is due to defective glucocerebrosidase, the enzyme responsible for the degradation of the glycosphingolipid glucocerebroside, a glucosylceramide. Three types of the disease have been described. Type 1 is the most common and lacks CNS involvement, while early onset neurological deterioration is present in types 2 and 3. Renal disease is rare, but glomerular deposition of glucocerebroside was described in mesangial and endothelial cells (Gaucher bodies), leading to severe proteinuria [84]. Farber disease is a lipogranulomatosis due to defective acid ceramidase β, which hydrolyzes ceramides into sphingosine and FAs and is coded by the *N*-acylsphingosine amidohydrolase (*ASAH1*) gene. Even if it is not clear whether there is ceramidase β activity in the human kidney, renal lipogranulomatosis due to ceramide accumulation was described in a patient with Farber disease [85]. Accordingly, in mice with podocyte-specific deletion of acid ceramidase β, the accumulation of ceramide in the glomeruli was shown, with consequent foot process effacement and nephrotic syndrome [86]. Mucolipidosis II and III are due to defective *N*-acetylglucosamine-1-phosphotransferase, which is involved in the modification of newly synthesized lysosomal enzymes with mannose-6-phosphate in the Golgi. Since this modification is key for protein delivery to the lysosomes, enzymes are released in the extracellular space, and their undigested substrates accumulate in the lysosomes. Rare cases of enlarged glomeruli with foamy podocytes full of glycolipids and foot effacement have been described. Lipid deposition was not detected in mesangial cells, endothelial cells, tubular cells, or interstitial cells, and renal function was not impaired [87, 88].

## Pharmacological correction of renal lipid imbalance

By correcting systemic dyslipidemia, lipid-lowering agents should decrease lipid uptake by renal cells; however, this effect does not always translate into a significant reduction of intrarenal lipid accumulation and modulation of CKD progression. For example, statins effectively reduce hypercholesterolemia and the incidence of cardiovascular events in CKD patients, but are not able to affect CKD progression, as shown by eGFR decline [89]. On the contrary, agents that are also able to correct intrarenal metabolic imbalance, as those activating AMPK, PPARα, FXR and LXRs or inhibiting the SREPBP pathway in podocytes and TECs, were shown to attenuate renal disease progression in animal models of CKD. Among these, PPARα agonists (fibrates and *n*−3 polyunsaturated FAs) modulated the expression of lipolytic genes, thus stimulating mitochondrial FAO and reducing SREBP activation; the consequent lipid clearance was associated with attenuation of renal disease progression in animal models of CKD due to diabetes, obesity, or mutations in the *PKD1* gene [70, 90–92]. Metformin, the most used glucose-lowering agent well-known for its ability to activate AMPK, ameliorated glycolipid metabolism and autophagy, reduced oxidative stress, and improved renal function in a diabetic rat model induced by high-fat diet and low dose streptozotocin [93]. The activation of the AMPK pathway is shared by the novel glucose-lowering agents, sodium-glucose cotransporter-2 (SGLT2) inhibitors and glucagon-like peptide 1 receptor agonists (GLP-1 RAs). The SGLT2 inhibitor empagliflozin reduced the accumulation of cholesterol and TG in diabetic and non-diabetic murine models of CKD through AMPK-mediated improvement of mitochondrial dynamics and function and reduction of aberrant glycolysis and SREBP-2 activation in TECs and podocytes [94, 95]. Similar findings were shown in diabetic and obesity-induced models of CKD treated with GLP-1 RAs [96, 97]. Historically, RAAS inhibitors have been used for their renoprotective effects based on blood pressure–independent restoration of intrarenal hemodynamics. However, recent evidence in animal models of DKD showed that ACE inhibitors and AT2 receptor antagonists alleviated renal injury also by promoting lipid clearance and restoring mitochondrial function through the modulation of the SREPBs and AMPK/PGC1α axes [98, 99]. Consistently, RAAS inhibitors ameliorated lipid abnormalities and kidney function in two patients with genetic LCAT deficiency [42, 100].

Among investigational drugs acting on novel targets, the dual agonist INT-767 for FXR (obeticholic acid) and the G protein–coupled receptor TGR5 reduced the accumulation of cholesterol, ceramides, and TG and decreased mesangial matrix expansion, podocyte loss, and renal fibrosis [101]. In animal models of DKD or Alport syndrome, promoting LD clearance by CE hydrolysis and cholesterol efflux through pharmacological inhibition or gene deletion of SOAT1 results in protection from disease progression [21]. Finally, the activation of transcription factor EB (TFEB), the master regulator of autophagy and lysosomal biogenesis, may rescue lysosomal dysfunction and the impaired autophagic flux observed in the kidneys of high fat–fed mice and obese patients [102, 103]. Interestingly, as suggested by recent evidence with the ATP citrate lyase inhibitor bempedoic acid, intrarenal lipid imbalance could be corrected also by the epigenetic modulation of lipogenic and fibrogenic gene expression. Indeed, the acetyl-CoA generated from citrate by ATP citrate lyase, whose activity is increased in the kidney of overweight/obese patients with CKD and in ob/ob mice, was shown to stimulate DNL not only by acting as a DNL substrate, but also by promoting histone acetylation [104].

## Common mechanisms of renal damage

The mechanisms through which lipid accumulation leads to renal cell apoptosis and fibrosis have not been fully elucidated. It is possible that lipotoxicity occurs by activating well-known and common molecular drivers of kidney disease, such as inflammation, oxidative stress, complement cascade, altered mitochondrial dynamics, and pro-fibrogenic signalling pathways (Fig. 2), which are described below.

These pathways have been extensively studied in podocytes, due to their key role in filtration, but are now investigated in tubular cells, as well. Indeed, while TECs were previously considered passive victims of injury, they are now recognized as active drivers of kidney disease progression through their pro-inflammatory and fibrogenic activity [105].

### Chronic inflammation

Chronic systemic inflammation triggers glomerulosclerosis, tubulointerstitial fibrosis, and tubular atrophy. It is due to the persistent activation of infiltrating immune cells, which release pro-inflammatory cytokines and chemokines that amplify inflammation and promote fibrosis. Interestingly, macrophages can exert a dual role, with M1 macrophages promoting early inflammation and tissue injury, while M2 ones contribute to tissue repair but also stimulate fibrosis by releasing pro-fibrotic factors like transforming growth factor-beta (TGF-β) and metalloproteinase inhibitors [106, 107]. Albuminuria and metabolic disturbances in CKD can trigger chronic inflammation, which over time accelerates kidney scarring and functional decline, increasing the risk of progression to end-stage renal disease (ESRD) [108]. Renal inflammation involves a complex interplay between different transcription factors, such as the nuclear factor κB (NF-κB), nucleotide-binding oligomerization domain-like receptor pyrin domain-containing 3 (NLRP3) inflammasome, and Janus kinase-signal transducer and activator of transcription (JAK/STAT).

NF-κB is a pivotal transcription factor in the inflammatory response associated with CKD. It regulates the expression of several pro-inflammatory factors through the binding to κB elements within promoter and enhancer regions. The NF-κB pathway also exhibits crosstalk with TGF-β/SMAD and mitogen-activated protein kinase (MAPK) signaling [109]. Under normal conditions, NF-κB is sequestered in the cytoplasm by inhibitors of κB (IκB) proteins. Inflammatory stimuli, as well as reactive oxygen species (ROS), angiotensin II (Ang II) or metabolites, such as glucose, can activate the IκB kinase (IKK) complex, leading to IκB degradation and nuclear translocation of NF-κB dimers [105, 110]. Additionally, epigenetic mechanisms, including microRNAs such as miR-21 and miR-802, can modulate NF-κB activity [111, 112].

The JAK-STAT signaling pathway is highly involved in renal inflammation and fibrosis. The pathway is activated by the binding of cytokines such as IL-6 and IFN-γ and their receptors, which undergo dimerization, JAK kinase recruitment, phosphorylation, and STAT activation. Phosphorylated STAT dimers translocate into the nucleus, where they enhance the transcription of pro-inflammatory and pro-fibrotic genes. Furthermore, STAT activation contributes to immune cell recruitment, while podocyte-specific JAK2 overexpression has been linked to the worsening of albuminuria, mesangial expansion, and glomerular damage in diabetic models [113, 114]. Persistent JAK-STAT activation has been especially observed in kidney diseases characterized by high inflammation, such as lupus nephritis, diabetic kidney disease (DKD), IgA nephropathy, and focal segmental glomerulosclerosis (FSGS) [113, 115, 116].

Inflammation can also be exacerbated by toll-like receptors (TLRs), which are overexpressed in injured renal cells, particularly TLR2 and TLR4. TLRs are activated by pathogen-associated molecular patterns (PAMPs) and by DAMPs, such as heat shock proteins and lipids; the consequent receptor dimerization and recruitment of adaptor proteins triggers downstream signaling pathways, including NF-κB and MAPK, promoting the production of inflammatory cytokines [105]. Additionally, TLR activation primes the NLRP3 inflammasome, enhancing oxidative stress and immune cell infiltration [117]. The JAK-STAT pathway also promotes TLR signaling by STAT-mediated upregulation of TLR expression [118].

NLRP3 is a cytosolic pattern recognition receptor that senses both DAMPs and PAMPs. Various stressors such as oxidative stress, mitochondrial DNA, potassium efflux, increased intracellular calcium, or lysosomal disruption trigger NLRP3 activation in renal cells and infiltrating immune cells. Upon activation, NLRP3 forms a multiprotein complex leading to the cleavage of pro-inflammatory IL-1β and IL-18 [105, 119]. Additionally, NLRP3 enhances NF-κB signaling and mitochondrial ROS generation [117]. Excessive NLRP3 activity has been implicated in DKD, hypertensive kidney injury, and obstructive nephropathy, while its inhibition has been shown to reduce renal inflammation and fibrosis [119, 120].

### Oxidative stress

Oxidative stress is characterized by an imbalance between the generation of ROS and the efficacy of endogenous antioxidant systems. The kidney, due to its high oxygen consumption, is particularly vulnerable to ROS-related damage. ROS are primarily generated by NADPH oxidases (NOX) and xanthine oxidase in the mitochondria. Among NOX family members, Nox4 is prominently upregulated in damaged renal cells through TGF-β/Smad, ERK1/2, and NF-κB activation [121]. Consistently, podocyte-specific Nox4 activation exacerbates glomerulosclerosis and albuminuria [122]. Excessive ROS can promote ER stress, hypoxia-inducible factor 1α (HIF-1α) upregulation, oxidative modifications of lipids, proteins, and DNA, and NF-κB activation, thus driving inflammation and fibrosis [123]. ER stress occurs when the ability of the ER to correctly fold newly synthesized proteins is exceeded; since the kidney has a high rate of protein synthesis, both glomerular and tubular cells are very sensitive to the effect of ER stressors [124].

### Mitochondrial dysfunction

Mitochondrial dysfunction is increasingly recognized as a key driver in the progression of both acute and chronic kidney diseases, especially in the presence of an ischemic injury. Hypoxia disrupts the electron transport chain, leading to excessive ROS production and cell damage [125]. Mitochondrial homeostasis is preserved through mitochondrial dynamics, mitophagy, and biogenesis. Indeed, mitochondrial fission and fusion are essential for maintaining mitochondrial integrity under stress conditions [126]; mitophagy selectively removes damaged mitochondria, while mitochondrial biogenesis, primarily regulated by PPARG coactivator 1α (PGC-1α), replenishes mitochondrial content, thus supporting cellular energy demands [127, 128]. Disruptions in these processes contribute to renal inflammation, cell apoptosis, and fibrosis. Proximal tubules, which are rich in mitochondria, are particularly vulnerable to mitochondrial dysfunction. In this context, several studies showed that increased PGC-1α levels enhanced mitochondrial mass, protecting the kidneys from ischemic and inflammatory injury, whereas its deficiency exacerbates renal dysfunction [129–132]. Moreover, it has been demonstrated that impaired mitochondrial dynamics increases apoptosis, inflammation, and renal fibrosis, while enhanced mitophagy exerts renoprotective effects by mitigating oxidative stress and inflammatory responses [133].

### Activation of the complement cascade

The complement system is a key player of innate immunity, conferring protection against pathogens. However, in several pathological conditions, the complement system is overactivated, leading to chronic inflammation and tissue injury. Persistent complement activation has been described in various forms of CKD, such as IgA nephropathy, FSGS, lupus nephritis, and DKD, where it triggers inflammation and fibrosis [117, 134–136]. The system consists of the systemic and local activation cascade of more than 50 different proteins by proteolysis. In the kidneys, podocytes and especially TECs can produce several complement components [105]. Among these proteins, the anaphylatoxins C3a and C5a bind to their G-protein–coupled receptors C3aR and C5aR1, activating pro-inflammatory and chemo-attractant pathways. Interestingly, increased renal expression of C3aR and C5aR1 and elevated plasma anaphylatoxin levels were described in immune and nonimmune-mediated forms of CKD and correlated with glomerular lesions and renal disease progression [117, 137]. Consistently, C3aR or C5aR1 blockade alleviated disease manifestations in animal models of CKD [138, 139]. Concomitantly, complement activation leads to the formation of the C5b-9 membrane attack complex, which forms a pore in the cell membrane, leading to cell lysis and death [137]. In podocytes, it disrupts cytoskeletal integrity and slit diaphragm function, leading to increased glomerular permeability and proteinuria [140]. In PTECs, where C3aR levels are particularly elevated, it promotes tubulointerstitial fibrosis by supporting epithelial-to-mesenchymal transition (EMT), RAAS activation, inflammation, TGFβ, and growth factor release [105, 141].

### Pro-fibrotic pathways

Renal fibrosis represents the final and irreversible pathological outcome of CKD, regardless of its cause. Chronic inflammation results in a maladaptive repair process leading to an excessive and deranged deposition of extracellular matrix (ECM) proteins, such as collagen and fibronectin, with progressive structural damage and organ dysfunction [142]. Recent studies suggest that fibrosis does not develop uniformly but rather originates in specific focal areas, called fibrogenic niches, where a complex interplay between local and infiltrated cells occurs [143]. Fibroblasts originate not only from resident stromal cells but also from TECs undergoing EMT, and from macrophages via macrophage-to-myofibroblast transition (MMT); indeed, myofibroblasts are largely absent in healthy kidneys, but extensively accumulate in injured kidneys contributing to ECM deposition [121, 144].

TGF-β is widely regarded as the most potent mediator of fibrosis in various organs, including the kidney. Among its three isoforms, TGF-β1 is the most relevant in kidney fibrosis and is produced by all renal cells. The binding of TGF-β1 to its receptors induces the nuclear translocation of Smad proteins which (i) enhance the transcription of pro-fibrotic genes and of plasmin activator inhibitor-1 that reduces ECM degradation, (ii) trigger monocyte/macrophage recruitment, and (iii) support EMT and MMT [121, 145, 146]. Beyond the canonical Smad signaling, TGF-β can also activate Smad-independent pathways, such as JNK, MAPK, PI3K/Akt, and Rho-like GTPases, all contributing to fibrosis through fibroblast activation and ECM deposition [147].

The Wnt/β-catenin pathway is another critical player in kidney fibrosis. Under normal conditions, β-catenin levels are tightly regulated by intracellular degradation. However, in renal disease, β-catenin is stabilized by the presence of Wnt ligands secreted by dysfunctional TECs; thus, β-catenin can translocate into the nucleus and promote the activation of fibroblasts and EMT [148]. The sustained activation of Wnt/β-catenin is also implicated in macrophage polarization, further worsening kidney injury [149]. A positive crosstalk exists between Wnt/β-catenin and TGF-β signaling in CKD, as β-catenin knockdown reduces TGF-β expression and fibrosis markers, while Wnt ligands directly induce TGF-β production [150].

The Notch signalling pathway is a highly conserved cell–cell communication system activated by the binding of Notch receptors with their ligands, promoting the transcription of pro-fibrogenic factors and of genes promoting EMT of TECs [121, 151]. The critical role in renal fibrosis is supported by the evidence that the inhibition of Notch signaling reduced fibrosis in experimental models of kidney disease [152]. Interestingly, Notch signaling also contributes to glomerulosclerosis, since Notch1 activation in podocytes and glomerular endothelial cells worsened proteinuria and structural damage [153].

Ang II is well known for its fibrogenic role in different organs, including the kidneys. Indeed, the binding of Ang II with its receptor type 1 (ATR1) stimulates the production of pro-fibrogenic factors supporting fibroblast activation and EMT and activates multiple signaling pathways as TGF-β/SMAD, MAPK, and NF-κB [154, 155]. TECs act as a local source of Ang II, which is further increased by high glucose conditions [156]. Aldosterone supports Ang II’s effect by increasing ATR1 expression and by promoting oxidative stress and NF-κB activation [157].

Finally, TECs are particularly sensitive to hypoxic conditions, which trigger fibrosis through HIF, by inducing a transcriptional reprogramming toward EMT, the activation of multiple pathways as NF-kB, TGF-β1/Smad, Wnt/β-catenin, Notch-1, and G2/M cell cycle arrest [158, 159].

## Conclusions and perspectives

Accumulating evidence indicates renal lipid accumulation in several forms of chronic kidney diseases of both metabolic and non-metabolic origin and supports an active role of lipids in the development and progression of the disease, even if causality is still to be proved. Since not all patients routinely undergo renal biopsy and samples are not usually processed for lipids, the prevalence and contribution of lipotoxicity to the pathogenesis of CKD could be underestimated; in vitro and in vivo studies on animal models may have a limited translational potential. Several lipid species have been involved and lipotoxicity can occur through various mechanisms. Indeed, besides their role as structural components of membranes and as energetic substrates, lipids could orchestrate several signaling cascades; most of them are well known for their role in CKD as triggers of inflammation, oxidative stress, organelle dysfunction, complement activation, apoptosis, and fibrogenesis. Improving our knowledge on the contribution of lipotoxicity to CKD is more than an academic exercise since many approaches to correct systemic or, more importantly, intracellular lipid imbalance are available or under investigation and could contribute to CKD management. In this context, some suggestions arise from available evidence. The first is that metabolism should always be addressed as a whole, as shown by the impact of high glucose on both systemic and intracellular lipid metabolism. The second is that correcting systemic dyslipidemia with lipid-lowering drugs could not effectively impact on CKD progression if intrarenal lipid metabolism is not affected as well, as for statins. In addition, research should move beyond addressing the effect of various stressors including lipids on glomerular cells and on tubular cells separately, but to consider the interconnections between these two compartments. Finally, even if the research on rare diseases is always challenging, it should always be promoted: besides providing useful insights to adequately monitor and manage affected patients, these diseases represent unique tools to generate extreme phenotypes, which could lead to the discovery of targets and mechanisms also relevant for common forms of renal disease.

## Data Availability

Data sharing is not applicable to this article, as no datasets were generated or analyzed during the current study.

## Abbreviations

*ALGS*: Alagille syndrome
*AMPK*: AMP-activated protein kinase
*Ang II*: Angiotensin II
*apoE*: Apolipoprotein E
*ASM*: Acid sphingomyelinase
*CE*: Cholesteryl esters
*CKD*: Chronic kidney disease
*DAMPs*: Damage-associated molecular patterns
*DKD*: Diabetic kidney disease
*DNL*: De novo lipogenesis
*ECM*: Extracellular matrix
*EMT*: Epithelial-to-mesenchymal transition
*ER*: Endoplasmic reticulum
*ESRD*: End-stage renal disease
*FABP*: Fatty acid-binding proteins
*FA*: Fatty acid
*FAO*: FA oxidation
*FATP*: FA transport proteins
*FSGS*: Focal segmental glomerulosclerosis
*FXRs*: Farnesoid X receptors
*GFR*: Glomerular filtration rate
*HDL*: High-density lipoproteins
*HIF*: Hypoxia-inducible factor
*IL*: Interleukin
*JAK/STAT*: Janus kinase-signal transducer and activator of transcription
*LCAT*: Lecithin:cholesterol acyltransferase
*LD*: Lipid droplets
*LDL*: Low-density lipoproteins
*LDL-R*: LDL-receptor
*LpX*: Lipoprotein X
*LRPs*: LDL-related protein receptors
*LSD*: Lysosomal storage disorders
*LXRs*: Liver X receptors
*MAPK*: Mitogen-activated protein kinase
*NF-κB*: Nuclear factor κB
*NLRP3*: Nucleotide-binding oligomerization domain-like receptor pyrin domain-containing 3
*NOX*: NADPH oxidase
*NPC*: Niemann–Pick C
*PAMPs*: Pathogen-associated molecular patterns
*PGC-1α*: PPARG coactivator 1α
*PL*: Phospholipids
*PPARα*: Peroxisome proliferator–activated receptor alpha
*PTEC*: Proximal TEC
*RAAS*: Renin-angiotensin-aldosterone axis
*ROS*: Reactive oxygen species
*SM*: Sphingomyelin
*SREBP*: Sterol regulatory element-binding protein
*TEC*: Tubular epithelial cell
*TCA*: Tricarboxylic acid
*TFEB*: Transcription factor EB
*TG*: Triglycerides
*TGF-β*: Transforming growth factor-beta
*TLR*: Toll-like receptor
*UC*: Unesterified cholesterol
